# Reactive oxygen species and fibrosis: further evidence of a significant liaison

**DOI:** 10.1007/s00441-016-2445-3

**Published:** 2016-06-27

**Authors:** Kati Richter, Thomas Kietzmann

**Affiliations:** Faculty of Biochemistry and Molecular Medicine and Biocenter Oulu, University of Oulu, Aapistie 7A, FI-90230 Oulu, Finland

**Keywords:** Reactive oxygen species (ROS), Antioxidants, Antioxidative enzymes, Matrix, Fibrosis

## Abstract

Age-related diseases such as obesity, diabetes, non-alcoholic fatty liver disease, chronic kidney disease and cardiomyopathy are frequently associated with fibrosis. Work within the last decade has improved our understanding of the pathophysiological mechanisms contributing to fibrosis development. In particular, oxidative stress and the antioxidant system appear to be crucial modulators of processes such as transforming growth factor-β1 (TGF-β1) signalling, metabolic homeostasis and chronic low-grade inflammation, all of which play important roles in fibrosis development and persistence. In the current review, we discuss the connections between reactive oxygen species, antioxidant enzymes and TGF-β1 signalling, together with functional consequences, reflecting a concept of redox-fibrosis that can be targeted in future therapies.

**Graphical abstract:**
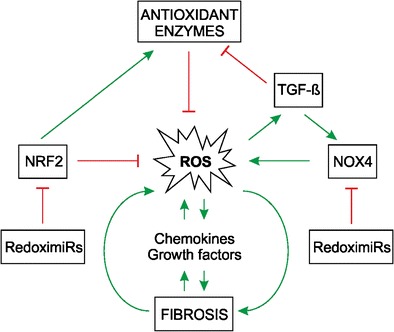
ᅟ

ᅟ

## Introduction

Age-related diseases and their associated complications such as organ fibrosis are considered to represent a major health problem worldwide. Fibrosis can be described as an irreversible non-physiological scarring process usually occurring as a consequence of inflammation or other injury in which an excessive appearance of extracellular matrix (ECM) contributes to further tissue damage. Fibrosis is not limited to any specific organ and can have multiple causes. For example, hepatic fibrosis can result from infection, alcohol, drugs or morbid obesity. Pulmonary fibrosis can be the result of radiation exposure or genetic alterations (e.g., cystic fibrosis) or it can be idiopathic. The various types of cardiac fibrosis range from congenital or idiopathic (e.g., endomyocardial fibrosis) to ischaemic. Retroperitoneal fibrosis and peritoneal fibrosis may result from therapies (the former has been associated with autoimmunity, malignancy and some forms of medication, such as β-blockers; the latter might be a complication of peritoneal dialysis). Many disorders, e.g., acquired, congenital, iatrogenic or allergic, culminate in renal fibrosis. Moreover, fibrosis of the skin is often caused by iatrogenic drug-related (eosinophilia-myalgia syndrome) or environmental (polyvinyl chloride, toxic/denatured “Spanish” rapeseed oil) factors (Boin and Hummers 2008). Thus, we need to understand the pathophysiology of fibrosis not only in the context of the wide spectrum of complications related to contemporary therapies and drugs but also as a consequence of chronic disease.

**Fig. 1:**
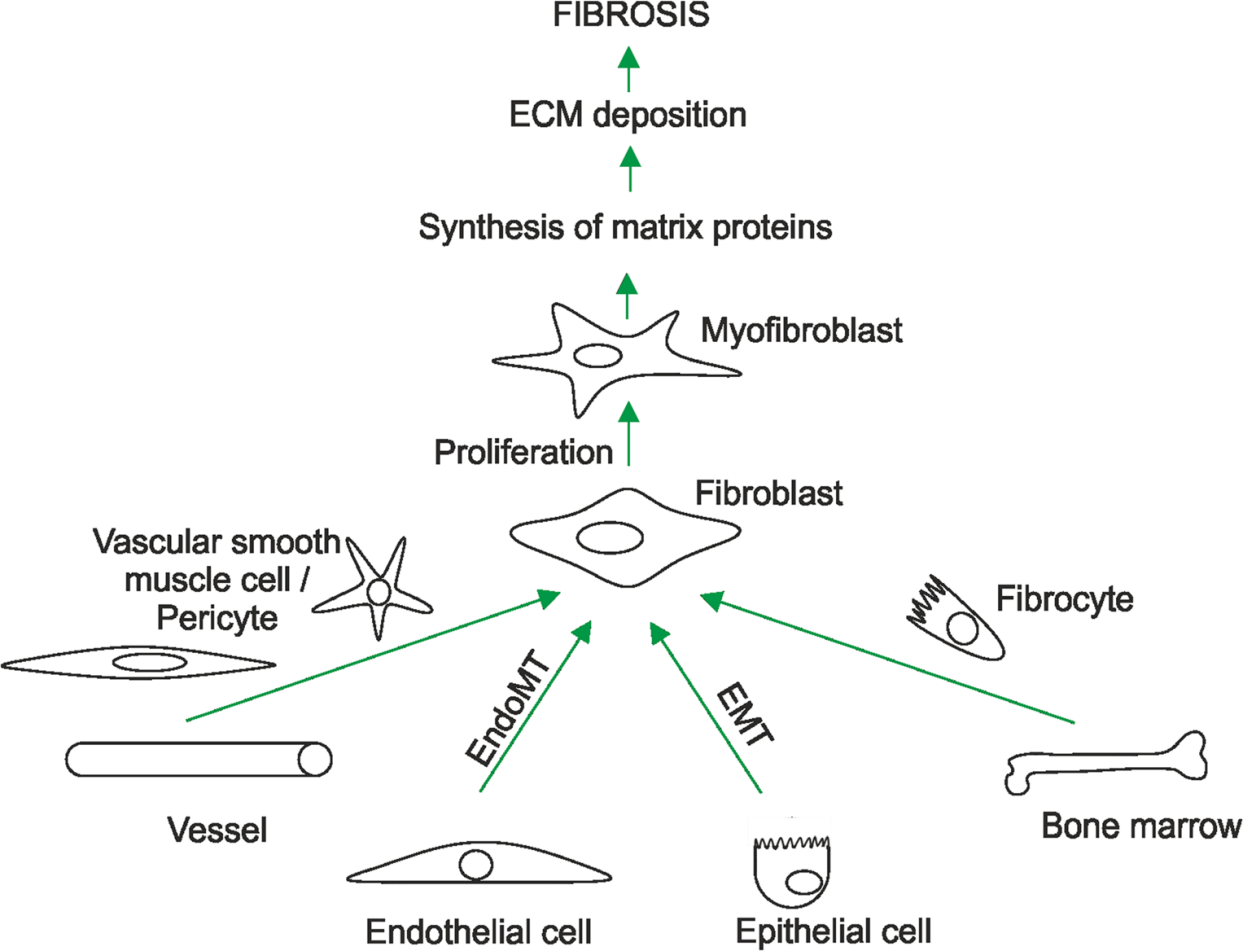
Activated fibroblasts are key players in the fibrotic process and extracellular matrix (*ECM*) production. Fibroblasts involved in fibrosis can be derived from vascular smooth muscle cells, pericytes, fibrocytes, endo- and epithelial cells or resident fibroblasts. Depending on their origin, these cells undergo proliferation, differentiation and endothelial/epithelial-to-mesenchymal transition (*EndoMT*/*EMT*) and the emerging myofibroblasts/activated fibroblasts excessively synthesize and secrete ECM proteins that contribute to fibrosis

Although some organ-specific aspects of fibrosis are known, fibrosis is not restricted to any particular organ and is found to occur in all organs and tumour tissues (Dvorak et al. 1984; Dvorak 1986; Kalluri and Zeisberg 2006; Rockey et al. 2015) suggesting the existence of a common pathogenetic pattern in which fibroblasts are primary ECM producers. Those fibroblasts possessing an increased synthetic capacity in fibrosis are called “myofibroblasts” or “activated fibroblasts” (Hecker et al. 2011). Myofibroblasts appear to be derived either from tissue-resident fibroblasts, bone-marrow-derived fibrocytes (Bucala et al. 1994) or vascular smooth muscle cells and pericytes shed from vessels (Lin et al. 2008; Ronnov-Jessen et al. 1995). In addition, endothelial cells that have undergone endothelial-to-mesenchymal transition (Zeisberg et al. 2007) and epithelial cells after epithelial-to-mesenchymal transition (Strutz et al. 1995) might give rise to fibrotic ECM production (Fig. 1).

A common pattern is also visible, macroscopically and microscopically. In addition to being hard and non-elastic, fibrotic tissue is also pale, reflecting injured parenchyma with an excess presence of fibrillar ECM and fibroblasts and a lack of capillaries. Further, a mononuclear infiltrate is also usually observed (Kalluri and Zeisberg 2006). In addition, recent findings at a more molecular level suggest that disturbances in the formation and degradation of reactive oxygen species (ROS) are a crucial part of the common fibrotic pathway. These findings have fed the concept of “redox-fibrosis”, which will be discussed in the current review together with its therapeutic potential.

## Fibrosis, ROS and oxidative stress

**Fig. 2:**
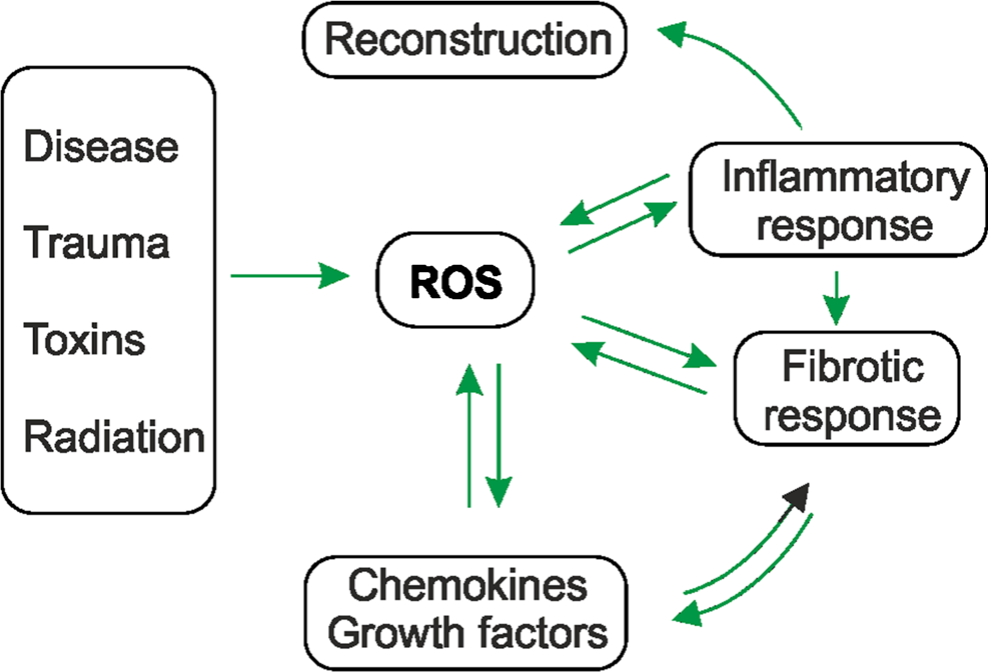
Reactive oxygen species (*ROS*) contribute to fibrosis via various feed-forward and feedback loops. Insults resulting from infectious diseases, trauma, toxins, drugs and radiation (UV, ionizing) induce ROS generation. Subsequently, ROS contribute to the fibrotic process either directly or indirectly via enhanced inflammation. Fibrosis and the inflammation itself might feedback into the pathway and further increase ROS formation or stimulate the production of cytokines and growth factors. The last two mentioned substances can also contribute to ROS formation. In a non-fibrotic process, inflammation and ROS formation end in tissue regeneration

Lost parenchyma after tissue injury is usually replaced, because of the ability of parenchymal cells to regenerate. However, the opportunity to regenerate usually becomes unavailable upon repetitive insults associated with chronic inflammation, the secretion of chemokines and the release of profibrotic metabolites, among them, ROS. Further, the production of ROS and the concomitant oxidative stress also contribute to the synthesis and activation of various cytokines and growth factors (Barnes and Gorin 2011; Babalola et al. 2014; Paik et al. 2014) indicating common feed-forward and feedback mechanisms (Fig. 2).

The link between ROS and oxidative stress became particularly evident from investigations showing that increased levels of hydrogen peroxide and oxidative stress markers such as 8-isoprostane were to be found in exhalants and in the urine of patients with lung fibrosis, respectively (Dekhuijzen et al. 1996; Pratico et al. 1998). In addition, enhanced levels of the lipid peroxidation marker 4-hydroxy-2′-nonenal (Seki et al. 2005) were found in biopsy specimens from patients with liver fibrosis. Moreover, ROS were proposed to be crucial for the development of asbestosis and silicosis, since nitrotyrosine adducts and the oxidative DNA damage indicator 8-hydroxy-2′-deoxyguanosine were found in those patients (Pilger et al. 2000; Pelclova et al. 2008). In addition, ROS and oxidative stress appear to be important in renal fibrosis (Ha and Lee 2003; Djamali et al. 2009) and tissue repair/remodelling after myocardial infarction (Murdoch et al. 2006; Sirker et al. 2007; Aragno et al. 2008).

### Sources and sites of ROS production

Ultraviolet light, ionizing radiation, toxic chemicals and drugs are well-known inducers of fibrosis and of ROS formation in a non-enzymatic manner in vivo (Fig. 2). Intracellularly, most ROS are by-products of the mitochondrial electron transport chain. In addition, ROS can be produced more specifically by various enzymes, among them members of the cytochrome P450 family, xanthine oxidoreductase, cyclo- and lipoxygenases, several peroxisomal oxidases and NADPH oxidases (NOX; for a review, see Samoylenko et al. 2013).

Of the ROS-producing enzymes, NOX appear to have a key role during fibrosis (Brown and Griendling 2009; O’Neill et al. 2015). The classic NOX from phagocytes forms a multiprotein complex, whereas the transmembrane proteins NOX2 (also known as gp91phox) and p22phox form the catalytic core for O_2_¯• production, which rapidly dismutates to hydrogen peroxide (Deffert et al. 2014; Nauseef 2014). In addition to NOX2, further NOX proteins designated NOX1–5 and the more distantly related DUOX1/2 proteins (Brown and Griendling 2009; O’Neill et al. 2015) have been identified. Although the expression of NOX2 is most evident in polymorphonuclear cells, macrophages and endothelial cells, its expression has also been verified in other cell types including cells from the central nervous system, smooth muscle cells, fibroblasts, cardiomyocytes, skeletal muscle, hepatocytes and haematopoietic stem cells (Gorlach et al. 2015). NOX1 is also present in the plasma membranes of various cell types (Brown and Griendling 2009; Aguirre and Lambeth 2010; Badid et al. 2010), whereas NOX3, NOX4 and NOX5 appear to have different subcellular locations and cell-specific expression patterns (for a review, see Gorlach et al. 2015). NOX3 is found in fetal tissues, the inner ear and lung endothelium and in HepG2 and RAW264.7 cells. NOX4 is widely expressed at very high levels in kidney, whereas NOX5 is more abundant in lymph nodes, spleen, prostate and testis and in endothelial and smooth muscle cells. Both NOX types appear to be mainly localized in the endoplasmic reticulum (for a review, see Gorlach et al. 2015). In general, NOX and DUOX activities, except those of NOX4, are controlled by regulatory subunits. Among these are the NOX2 cytosolic subunits p40phox, p47phox and p67phox, their NOX1- and NOX3-regulating homologues NOXO1 and NOXA1 and the DUOX1/2 regulators DUOXA1 and 2 plus the GTPase Rac. In addition, the activation of NOX5 and DUOX1/2 is dependent on calcium signalling (BelAiba et al. 2007). NOX4 activity seems to be independent of regulatory subunits.

NOX-derived ROS have been found to be associated with fibrosis in several organs such as lung (Hecker et al. 2009), heart (Cucoranu et al. 2005), kidney (Sedeek et al. 2013), pancreas (Masamune et al. 2007) and liver (De Minicis and Brenner 2007; Cui et al. 2011; Paik et al. 2011). Of the NOX proteins, NOX4 is unique in that its activity is mainly regulated via its expression levels and that it does not require further regulatory subunits except for its dimerization partner p22phox (Petry et al. 2006; Paik et al. 2014). Moreover, NOX4 has been closely linked to endothelial cell dysfunction and hypoxia, conditions known to promote its expression (Bernard et al. 2014). Thus, compared with the more complex regulation of the other NOX enzymes, NOX4 might have a more critical role in ROS production under conditions promoting fibrosis.

## ROS and TGF-β are interlinked by feed-forward and feedback mechanisms

The onset and progression of fibrosis appear to involve not only ROS formation but also the synthesis and secretion of various growth factors and chemokines. Cytokines of the transforming growth factor-β (TGF-β) family crucially participate in the fibrotic process in most if not all organs (Akram et al. 2013; Aravinthan et al. 2013; Rudolph et al. 2000). So far, three TGF-β isoforms (called TGF-β1, TGF-β2 and TGF-β3) have been identified; before these isoforms were discovered, TGF-β referred only to TGF-β1. Although primarily linked to fibrosis, TGF-β1 also seems to be important in the pathogenesis of other diseases such as Marfan syndrome, Parkinson’s disease, various forms of cancer and diabetes (for reviews, see Leask and Abraham 2004; Leask et al. 2004; Zheng 2009). Importantly, the production of TGF-β1 is increased in all fibrotic tissues. Further, it can induce collagen expression in fibroblast cell cultures of various origins (Romanelli et al. 1997; Kim et al. 1998; Abraham et al. 2000; Hogaboam et al. 2000). Moreover, increased production of ROS and oxidative stress are connected to TGF-β1 activation and production, indicating that they are key to the fibrotic process (Barnes and Gorin 2011; Radwan et al. 2012).

### TGF-β1 is involved in ROS production

In various cell types, ROS formation has been shown to be enhanced in response to TGF-β1. This process appears to be predominantly a consequence of the TGF-β1-mediated induction of NOX4 expression and its subsequent increased activity (Cucoranu et al. 2005; Sturrock et al. 2006; Carmona-Cuenca et al. 2008; Boudreau et al. 2012). Likewise, NOX4 is selectively up-regulated in the lungs of patients with idiopathic pulmonary fibrosis and is associated with endothelial cell dysfunction and hypoxia, two processes that can foster the further up-regulation of NOX4 expression (Diebold et al. 2010a, 2010b). In addition, short interfering RNA (siRNA)-mediated NOX4 knockdown inhibits TGF-β1-mediated pro-fibrotic responses and ECM deposition in the lungs of mice (Hecker et al. 2009). Further, NOX4 knockout mice are more protected against bleomycin-induced acute lung injury and the onset of fibrosis (Carnesecchi et al. 2011) than NOX2 knockout mice (Manoury et al. 2005). In agreement with this, the use of a low-molecular-weight NOX4 inhibitor in mice attenuates the bleomycin-induced pulmonary fibrosis (Jarman et al. 2014). In addition, NOX4 has also been found to be increased in patients with hepatitis C virus (HCV)-associated liver fibrosis and in patients with non-alcoholic steatohepatitis (Bettaieb et al. 2015). Furthermore, hepatic stellate cells (HSCs), which have a key role during liver fibrosis, induce NOX4-dependent ROS formation upon stimulation with TGF-β1 (Proell et al. 2007) and the down-regulation of NOX4 by siRNA or the absence of NOX4 in mice inhibits the TGF-β-induced fibrotic process (Sancho et al. 2012). Although these studies indicate a dominant role of NOX4 in fibrosis, other investigations also indicate roles of NOX1 and NOX2 (Imura et al. 1992; Aram et al. 2008, 2009; J.X. Jiang et al. 2010; Paik et al. 2011).

In agreement with the consideration that fibrosis is an age-related disease, NOX4 and its role in fibrosis have also been found to be connected with aging. In particular, NOX4 appears to have different roles in young and in aged mice; in young mice, NOX4 stimulates myofibroblast differentiation and wound healing but, in aged mice, it induces fibrosis (Hecker et al. 2009, 2014; Thannickal 2010). The fibrotic response in aged animals might be the result of a reduced antioxidant response. Accordingly, the ratio between NOX4 and the antioxidant transcription factor NRF2 (nuclear factor erythroid 2-related factor 2; see below) has been proposed to be crucial for the development of fibrosis and apoptosis-resistant myofibroblasts in aged mice (Thannickal 2010). Together, TGF-β1-driven ROS formation involving NOX4 appears to play an important role in the pathogenesis of fibrosis, in particular, in elderly subjects.

### TGF-β1 is a negative regulator of the antioxidative response

**Fig. 3:**
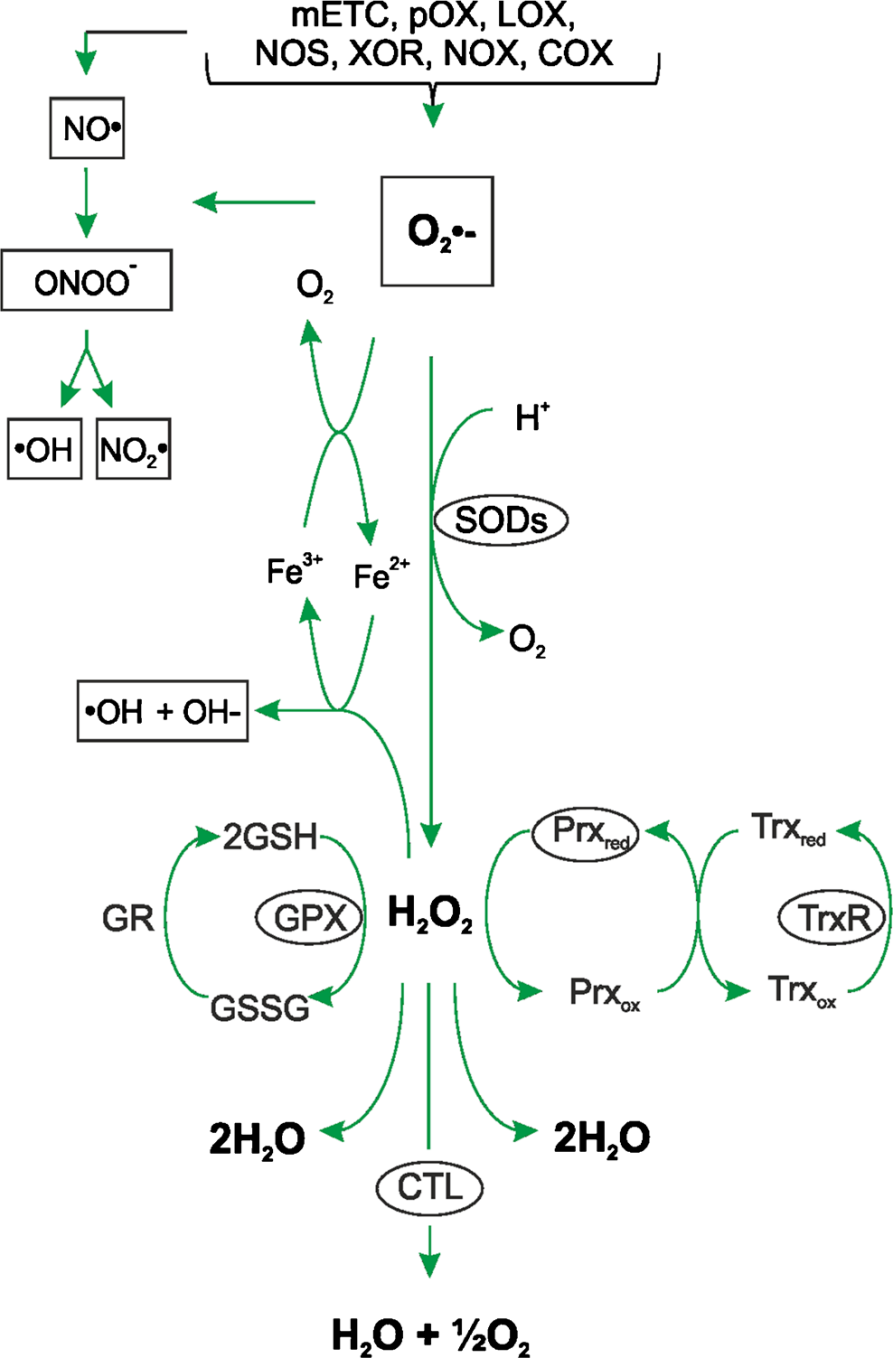
Various antioxidant enzymes act on ROS. Superoxide (*O*_*2*_^*•-*^) is a major ROS and can be produced by various enzymes; it serves as the main precursor for the production of other ROS and, upon reaction with nitric oxide (*NO* ^*•*^), it gives rise to peroxynitrite formation (*ONOO* ^*−*^). Antioxidant enzymes (*ellipsoids*) such as superoxide dismutase (*SOD*) are able to convert O_2_^•-^ to hydrogen peroxide (*H*_*2*_ *O*_*2*_), which can be neutralized through the action of glutathione peroxidases (*GPX*), peroxiredoxins (*Prx*) or catalase (*CTL*). Non-neutralized hydrogen peroxide may be converted in the presence of Fe^2+^ into hydroxyl radicals (^*•*^ *OH*) and hydroxyl anions (*OH* ^*−*^) in the Fenton reaction (*mETC* itochondrial electron transport chain, LOX lipoxygenase, pOX peroxidase, *NOX* NADPH oxidase, *XOR* xanthine oxidoreductase, *COX* cyclooxygenase, *NOS* nitric oxide synthase, *NO*_*2*_^*●*^ nitrogen dioxide, *Trx* thioredoxin, *TrxR* thioredoxin reductase, *GR* glutathione reductase, *GSH* glutathione, *GSSG* oxidized glutathione)

The increase in ROS levels in response to TGF-β1 may also be a result of the suppressed expression of several antioxidant enzymes. As a result, the formation and removal of ROS are no longer balanced leading to oxidative stress. A number of exogenous substances and endogenous molecules, among them glutathione (GSH) and several enzymes, such as superoxide dismutases (SODs), glutathione peroxidases (GPXs) and catalase (CTL) represent major players in the antioxidant defence system (Samoylenko et al. 2013; Fig. 3).

The ubiquitously present reduced three-residue peptide GSH (γ-L-glutamyl-L-cysteinyl glycine) acts as a major cellular antioxidant (Foyer and Noctor 2011). It is present at a relatively high concentration (1–10 mM) and is able to donate an electron, a process upon which two molecules of GSH form oxidized glutathione (GSSG). This process is reversible and is carried out by the enzyme glutathione reductase (Fig. 3). Although GSH can directly react with O_2_•¯ and some other ROS, its indirect ROS-scavenging functions are more important (Winterbourn and Metodiewa 1994; Winterbourn 2008; Blokhina and Fagerstedt 2010). In particular, GSH can regenerate other antioxidants, e.g., it can reduce α-tocopherol radicals and semihydroascorbate radicals (Blokhina and Fagerstedt 2010) or, together with GPXs, can convert hydrogen peroxide into water. Further, GSH acts together with glutaredoxins and glutathione S transferase (GST) to detoxify reactive electrophilic compounds, which are products of oxidative stress and often constituents of environmental toxins (Fernandes and Holmgren 2004).

Patients with fibrotic diseases such as liver cirrhosis, viral hepatitis, chronic obstructive lung diseases and asbestosis display reduced GSH levels, which may contribute to increased ROS levels (Gao and Bataller 2011; Geybels et al. 2013; Choi et al. 2014). One factor that might be of importance in the pathogenic process of fibrosis is chronic alcohol abuse, which is well known as being a major reason for chronic liver disease and cirrhosis, whereby it can cause an 80–90 % depletion of GSH in the liver (Bianchi et al. 2000). However, GSH restriction because of alcohol consumption appears not to be restricted to liver tissue, as it can also occur in the lungs (Bianchi et al. 2000). A major reason for the GSH deficiency appears to be decreased GSH generation triggered by TGF-β1, which affects the expression of one of the gamma-glutamylcysteine synthetase (gamma-GCS) subunits. Gamma-GCS is the rate-controlling enzyme of GSH synthesis (Arsalane et al. 1997; Ramani et al. 2012) and consists in the regulatory light (gamma-GCSl) subunit and the catalytic heavy (gamma-GCSh) subunit. Of these two subunits, TGF-β1 has been shown to down-regulate the expression of gamma-GCSh in the fibrotic areas of interstitial pneumonia (Tiitto et al. 2004) and in human lung alveolar epithelial cells (Liu et al. 2012). The reduction of gamma-GCSh expression and GSH generation in response to TGF-β1 is also in agreement with the increased protein oxidation and lipid peroxidation in mice with lung fibrosis (Liu et al. 2012). All in all, TGF-β1-mediated reduction in GSH production might contribute to oxidative stress during the fibrotic process.

As mentioned above, GSH can act together with the glutaredoxins (Grxs), which function as thiol-disulphide oxidoreductases. In connection with this, TGF-β1 has been shown to reduce Grx1 expression, again indicating that GSH-driven processes are of importance in fibrosis (Peltoniemi et al. 2006; Harju et al. 2007).

Another family of enzymes with antioxidant functions are the SODs, which catalyse the conversion of O_2_•¯ into hydrogen peroxidase and O_2_. Mammals possess three SODs: cytosolic (SOD1), mitochondrial (SOD2) and extracellular (SOD3) variants. Although the generated hydrogen peroxidase can be converted into oxygen and water by catalase (Samoylenko et al. 2013; McCord and Fridovich 2014), it appears that peroxiredoxins (Prxs) are more important, since they react with hydrogen peroxidase at an exceptionally high rate (Wood et al. 2003; Fig. 3). In particular, Prx2 is considered to trap almost all hydrogen peroxidase in vivo (Winterbourn 2008) as a result of its high abundance and reaction rate. Moreover, Prx members not only react with hydrogen peroxidase, but also with peroxynitrite and other organic hydroperoxides. Importantly, the various Prx members are localized in different cellular compartments, with Prx1, 2 and 4 occurring mainly in the cytoplasm and nucleus. In addition, Prx1 can also be found in mitochondria and peroxisomes, whereas Prx4 has been located in lysosomes (Oberley et al. 2001; Immenschuh et al. 2003; Go and Jones 2010). Prx3 is present in mitochondria, like Prx5, which can also be found in the cytoplasm, nucleus and peroxisomes (Seo et al. 2000). Once oxidized, Prx molecules are reduced by thioredoxins (Trxs). The Trx proteins themselves are subject to oxidation–reduction reactions, whereby various stimuli can contribute to their oxidation and thioredoxin reductase (TrxR) mediates their NADPH-dependent reduction (Holmgren et al. 2005; Fig. 3). Again, these systems are compartmentalized, with Trx1 and TrxR1 in the cytoplasm and nucleus and Trx2 and TrxR2 in the mitochondria (Oberley et al. 2001; Go and Jones 2010).

In addition to the Prx members, GPXs are also able to reduce peroxides in various compartments. The ubiquitous member is GPX1, which reduces peroxides mainly in the cytosol, mitochondria and peroxisomes. GPX2 acts in epithelia and its highest levels are found in the intestine (Lubos et al. 2011). Another variant is represented by the secreted GPX3, which is found predominantly in lung and kidney and protects against peroxides arising from outside the cell (Lubos et al. 2011). Three isoforms of GPX4, namely the cytosolic (c-GPX4), mitochondrial (m-GPX4) and nuclear (n-GPX4) isoforms (Nomura et al. 2000; Imai and Nakagawa 2003), which are derived from a single gene, have been identified so far. In addition to hydrogen peroxide, GPX4 substrates include derivatives of cholesterol and cholesteryl esters and thymine hydroperoxide (Imai and Nakagawa 2003).

The usefulness of having several systems for hydrogen peroxide conversion is mainly for preventing iron-catalyzed Fenton reactions and the subsequent generation of highly reactive hydroxyl radicals (•OH; Fig. 3). In addition, iron-binding proteins, such as ferritin and transferrin, which sequester free iron, also contribute to this effect. Further, so-called “dietary micronutrients” such as vitamin C, vitamin E and selenium also participate in the antioxidative process (Samoylenko et al. 2013).

Interestingly, the roles of the antioxidant enzymes during fibrosis are not yet well known. However, TGF-β1 appears to act as a negative regulator and inhibits mRNA expression and the activities of GPX1 and CTL in a hamster pancreatic beta-cell line (HIT). Concomitantly, an increase in ROS levels and oxidized proteins can be detected (Islam et al. 1997). In addition, TGF-β1 also decreases SOD1, SOD2, CTL and GST expression and increases cellular ROS levels in cultured rat hepatocytes and airway smooth muscle cells (Kayanoki et al. 1994; Islam et al. 1997; Michaeloudes et al. 2011).

Together, the fibrotic action of TGF-β1 appears to be coupled to the suppressed expression of antioxidant enzymes. This subsequently leads to the increased production of ROS because of the prevailing action of ROS-generating systems.

## Fibrosis and NRF2 signalling

The transcription factor NRF2 and its binding partner Keap1 (Kelch-like ECH-associated protein 1) regulate the transcription of various antioxidant enzymes (Hayes and Dinkova-Kostova 2014; Levonen et al. 2014). In the absence of ROS, Keap1 is bound to NRF2 and promotes its proteasomal degradation. The sulphhydryl groups at the cysteine residues in Keap1, with Cys151 as the most critical, are able to mediate redox sensitivity (Kensler et al. 2007; Hayes and Dinkova-Kostova 2014). They become oxidized upon an increase in ROS and Keap1 loses its binding to NRF2. Moreover, ROS cause the dephosphorylation of Keap1 at Tyr141, an event that promotes Keap1 degradation (Jain et al. 2008). As a consequence, NRF2 can no longer be degraded and is transported to the nucleus, where its presence is promoted because of the inability of the nuclear export protein CRM1 to bind the Cys183-oxidized NRF2. In the nucleus, NRF2 heterodimerizes with a small Maf protein and activates genes whose products are involved in the antioxidant response (Kensler et al. 2007; Hayes and Dinkova-Kostova 2014).

Several investigators have pointed out that NRF2 signalling is of importance in the pathogenesis of fibrosis. Indeed, NRF2^−/−^ mice have been found to be more prone to chemically induced oxidative stress than wild-type mice (Aleksunes and Manautou 2007; Liu et al. 2013). This is particularly evident in the liver, where NRF2 protects mice from carbon-tetrachloride-induced hepatic fibrosis (Xu et al. 2008) or fibrosis when fed a methionine- and choline-deficient diet (Chowdhry et al. 2010; Sugimoto et al. 2010; Zhang et al. 2010; Okada et al. 2012). In addition, the response to high-fat diets or chronic alcohol abuse appears to involve the suppression of NRF2. Indeed, the onset of bleomycin-induced lung fibrosis in mice has been found to be primed by chronic alcohol abuse; the priming effect is considered to be the result of the reduced expression of the NRF2-dependent genes for GST theta 2 and the catalytic subunit of glutamate-cysteine ligase and of the increased expression of TGF-β1 (Sueblinvong et al. 2014a). The increased TGF-β1 expression upon alcohol ingestion in this model has subsequently been shown to be the dominant modulator, since the blocking of the TGF-β1 signal attenuates the alcohol-induced suppression of NRF2 (Sueblinvong et al. 2014b). Moreover, and in agreement with fibrosis being an age-related disorder, NRF2-mediated protection against chemically induced fibrosis has been found to be less efficient with aging (Aravinthan et al. 2013).

The NRF2/Keap1 system is also subject to further modulation by other regulatory cascades. For instance, the selenoprotein TrxR1 has been found to regulate NRF2; consequently, a deficiency of selenium and the concomitant loss of TrxR1 activity not only affect NRF2 but are also combined with the induction of NOX activity and oxidative stress (Cebula et al. 2015). Moreover, the transcription factor Krüppel-like factor 9 (Klf9) can be induced by ROS via NRF2. This is an important aspect of the pathogenesis of fibrosis, since Klf9 has been shown to increase ROS in vitro and in vivo in mice and to promote bleomycin-induced pulmonary fibrosis (Zucker et al. 2014). Thus, the induction of Klf9 by NRF2 results in a critical feed-forward response that might promote ROS formation and fibrosis.

A number of substances naturally occurring in plants, such as quercetin, genistein and curcumin, are considered to be health-beneficial. They are, among environmental agents such as paraquat, metals and endogenous substances such as hydrogen peroxide, NO and 4-hydroxynonenal, known to be NRF2 activators (Ma and He 2012). Although this implies that the induction of NRF2 is of therapeutic benefit, NRF2 activation attributable to Keap1 absence in mice has been found to cause death shortly after birth, although the lethal phenotype can be rescued in Keap1/NRF2 double-knockout mice (Wakabayashi et al. 2003). Another study has shown that the constitutive activation of NRF2 promotes tumour survival (T. Jiang et al. 2010). These findings indicate that chronic or excessive NRF2 activation negatively affects cellular behaviour and normal life span.

Overall, the NRF2 pathway appears to be critically involved in the ROS-mediated regulation of fibrosis, especially in connection with its inactivation in response to TGF-β1. However, the cross-talk and feed-forward and feedback mechanisms impacting the NRF2 system are not yet completely understood and thus remain to be resolved in order to gain a more complete picture of its role in fibrosis.

### ROS activate TGF-β

**Fig. 4:**
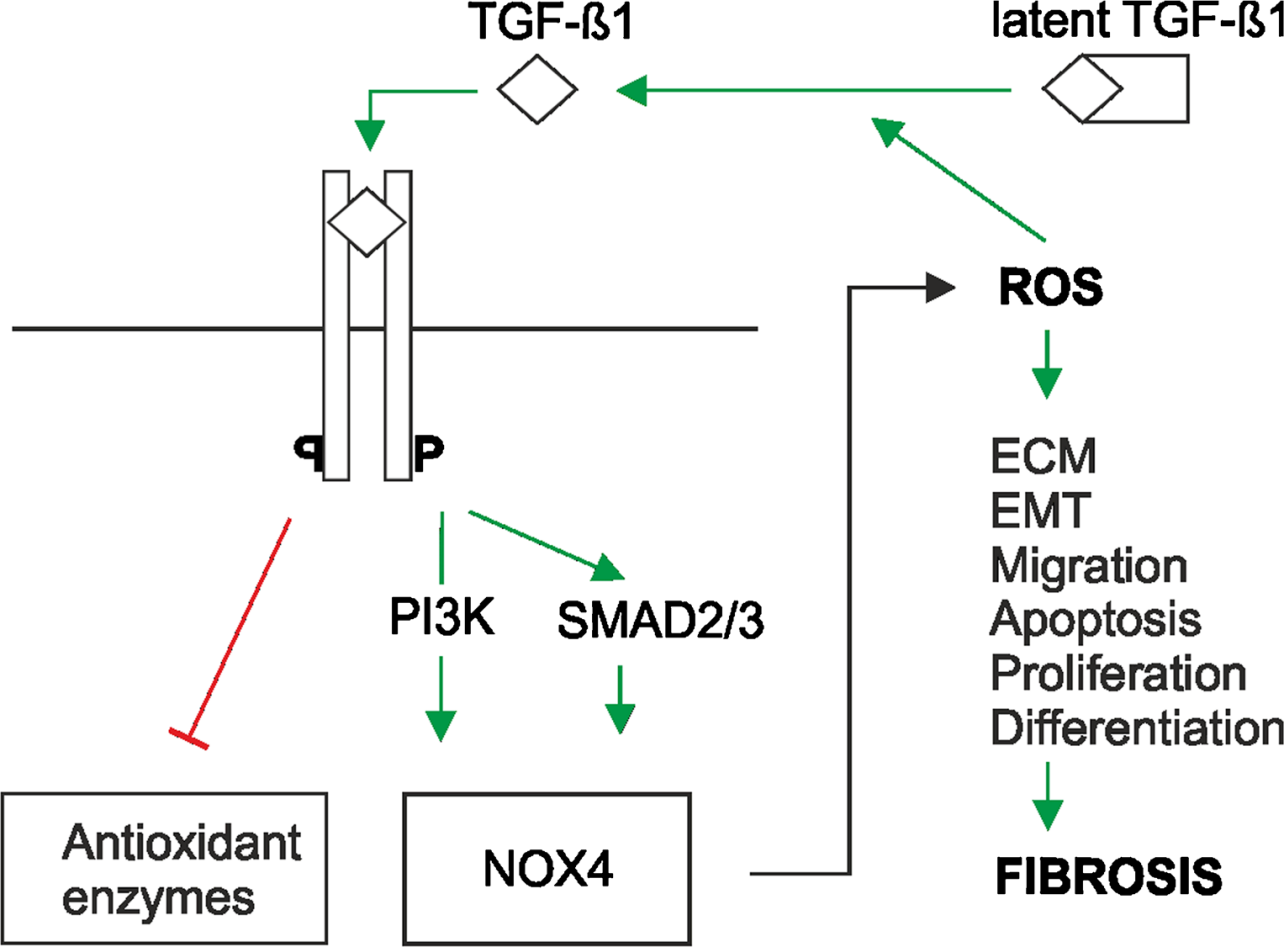
ROS are involved in transforming growth factor-β (TGF-β)-mediated fibrosis. Various cell types such as platelets, parenchymal cells and inflammatory cells (e.g., lymphocytes, macrophages) can release TGF-β1. After conversion of the latent to the active form, TGF-β1 binds to its receptor and induces SMAD2/3 and/or phosphatidyl inositol 3-kinase (*PI3K*) signalling to express various genes, among them that for NOX4. NOX4 in turn leads to ROS production. Enhanced ROS may activate the proliferation, migration and differentiation of fibroblasts plus epithelial-to-mesenchymal transition (*EMT*), apoptosis of epithelial cells and/or excessive extracellular matrix (*ECM*) deposition. In addition, TGF-β1 contributes to ROS production by attenuating the expression of antioxidant enzymes such as glutaredoxin, catalase, glutathione peroxidase, glutathione S transferase, superoxide dismutase and the heavy subunit of gamma-glutamylcysteine synthetase

Like many growth factors and hormones, TGF-β needs to be activated. Normally, TGF-β is bound to two polypeptides: a latent TGF-β-binding protein (LTBP) and a latency-associated peptide (LAP). Together, they form an inactive, so-called latent TGF-β complex. To become active, TGF-β needs to be released from this complex (Fig. 4). This can be achieved via serum proteases such as plasmin, a number of matrix metalloproteases and thrombospondin-1 (Shi et al. 2011; Robertson et al. 2015). Furthermore, integrins, pH and ROS are able to activate TGF-β (Lyons et al. 1988; Munger et al. 1998; Jobling et al. 2006), although whether this activation occurs directly or indirectly via integrin-, pH- or ROS-mediated activation of proteases is not resolved.

With respect to ROS, the activation of TGF-β1 appears to be direct, since ROS generated in a cell-free system by ionizing radiation or metal ion-catalyzed ascorbate reactions in solution can activate recombinant latent TGF-β1 (Jobling et al. 2006). In particular, TGF-β1 activation in response to metal ion-catalyzed ascorbate oxidation is very efficient and has been shown to depend on transition metal ions and ascorbate. Further, the ROS-dependent activation occurs only with TGF-β1 and not the other isoforms; this has been found to be attributable to ROS-mediated oxidation of the LAP-beta1 protein at methionine 253 (Jobling et al. 2006). All in all, enhanced ROS production can be crucial for the activation of TGF-β1, indicating another causal link to fibrosis.

## Fibrosis and ROS are linked to hypoxia

The fibrotic process is not only characterized by enhanced ROS levels but is also associated with hypoxia attributable to the loss of endothelial cells and the rarefication of capillaries. The reduction of endothelial cells is largely caused by endothelial-mesenchymal transition (EndoMT), a process whereby endothelial cells undergo transformation and acquire a mesenchymal (fibroblast-like) phenotype that allows these cells to migrate and to acquire invasive properties; a similar process called epithelial-mesenchymal transition (EMT) can occur with epithelial cells. Although EndoMT and EMT are usually dormant in adult organs, insults, inflammation or chronic diseases can reactivate these embryonic processes (Dimmeler and Zeiher 2004; Abraham and Varga 2005; Asada et al. 2011).

The transcription factors hypoxia-inducible factor-1α (HIF-1α) and hypoxia-inducible factor-2α (HIF-2α) appear to integrate EMT, fibrosis and the responses to various stimuli at the level of ROS signalling (Lavrovsky et al. 2000; Gorlach et al. 2015). Indeed, preclinical and clinical studies show that the fibrotic process correlates with the expression of hypoxia-inducible HIF-1α target genes such as those for tissue-inhibitor of metalloproteinases-1, plasminogen activator inhibitor-1 (PAI-1) and connective tissue growth factor (Kaminski 2006; Tzouvelekis et al. 2007). Further, data from pulmonary fibrosis mouse models and idiopathic pulmonary fibrosis patients have revealed increased HIF-1α expression in alveolar epithelial cells at an early stage of pulmonary fibrosis (Tzouvelekis et al. 2007). Moreover, TGF-β1 and hypoxia signalling appear to undergo mutual interactions, since the major TGF-β-responsive transcription factor Smad3 can be up-regulated by hypoxia (Zhang et al. 2003) and, vice versa, the induction of type I collagen expression by TGF-β1 can be decreased upon inhibition of HIF-1 (Basu et al. 2011).

TGF-β and hypoxia are also drivers of EMT. Although hypoxia via HIF-1α (Distler et al. 2004; Moon et al. 2009) promotes the expression of endothelial growth factors to improve the capacity to regenerate vessels (or form new ones), the link to EMT suggests that fibroblast formation and EMT will occur (Kim et al. 1998). Indeed, hypoxia and the stable expression of HIF-1α have been associated with increased renal fibrosis and EMT, whereas the injection of vascular endothelial growth factor (VEGF) is beneficial in some experimental models of organ fibrosis (Corpechot et al. 2002; Yoon et al. 2005; Ioannou et al. 2013). In agreement with this evidence, the loss of HIF-1α in primary renal epithelial cells reduces EMT and the targeted deletion of HIF-1α in proximal tubular epithelial cells reduces tubulointerstitial fibrosis upon unilateral urethral obstruction (Higgins et al. 2007). Interestingly, the transdifferentiation of kidney tubular epithelial cells into myofibroblasts appears to be a result of HIF-mediated regulation of stromal cell-derived factor 1 (SDF-1) and its receptor CXCR4 (Barriga et al. 2013), whereas a recent study of human coronary endothelial cells demonstrated that HIF-1α-driven expression of the transcription factor Snail is responsible for EMT (Xu et al. 2015). Together, these data suggest that hypoxia has opposing effects and, probably depending on the regenerative capacity of the injured tissue, can contribute to vessel regeneration via HIF-driven VEGF expression or to the progression of fibrosis via EMT.

## Fibrosis is affected by ROS via post-transcriptional and epigenetic mechanisms

Knowledge concerning the pathogenesis of fibrosis has improved greatly, although a large number of issues are still not resolved. Among these is the often occurring inter-individual difference in the severity and progression of fibrosis. Recent advances in understanding post-transcriptional gene-regulation events, genetic variability and epigenetic phenomena have suggested that these mechanisms contribute to the variability often seen in individuals with fibrosis (Wynn 2010).

**Fig. 5:**
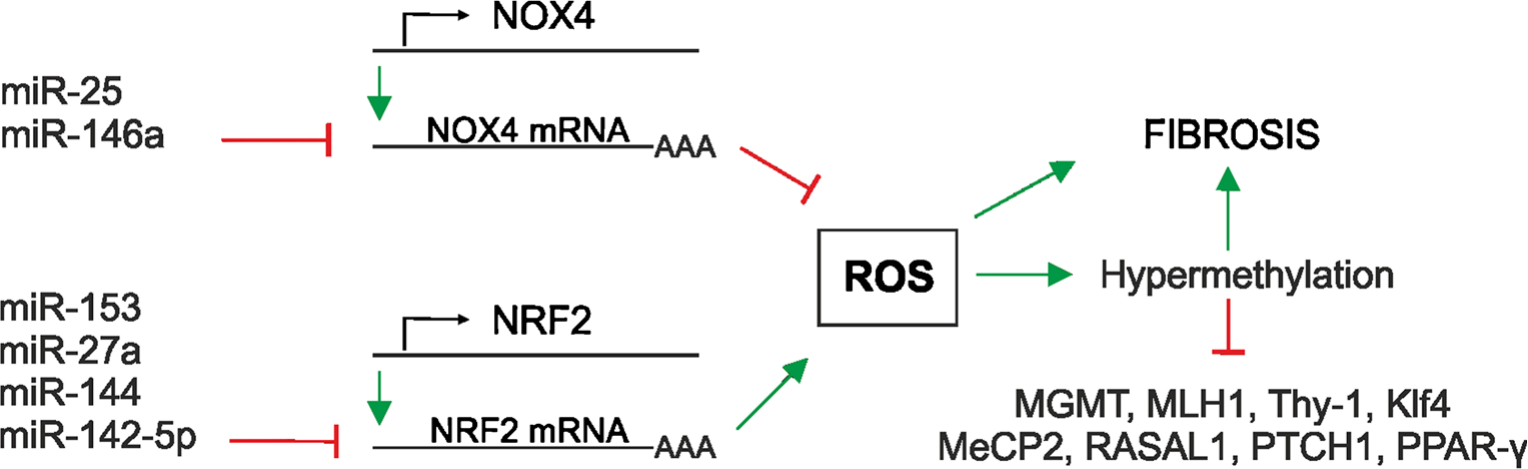
Fibrosis and ROS are interconnected with microRNA (miRNA) expression and epigenetic modifications. Several miRNAs affect cellular ROS levels via the post-transcriptional degradation of NOX4 and nuclear factor erythroid 2-related factor 2 (*NRF2*) mRNA. In addition, epigenetically, ROS-associated DNA hypermethylation contributes to the reduced expression of various genes, among them O-6-methylguanine-DNA methyltransferase (*MGMT*), mutL homologue 1 (*MLH1*), thymocyte differentiation antigen-1 (*Thy-1*), Krüppel-like factor 4 (*Klf4*), methyl CpG binding protein 2 (*MeCP2*), RAS protein activator 1 (*RASAL1*), peroxisome proliferator-activated receptor (*PPAR-γ*) and patched1 (*PTCH1*)

Post-transcriptional regulation is largely achieved by mRNA degradation and translational repression, processes in which microRNAs (miRNAs) play a crucial role. Indeed, several miRNAs have been found to be regulated by TGF-β1 and shown to be regulators of pro- and anti-fibrotic processes (Pottier et al. 2014). The connection between ROS, fibrosis and miRNA expression is underlined by findings showing that TGF-β1 regulates NOX4 expression and the NOX4-dependent generation of hydrogen peroxide, which has been found to be required for TGF-β-induced myofibroblast differentiation, ECM production and contractility (Hecker et al. 2009). The expression of NOX4 and thus ROS generation can be down-regulated by the so-called “redoximiRs” miR-146a and miR-25 (Cheng et al. 2013), whereas miR-135b and miR-708 can be up-regulated by hydrogen peroxide (Fig. 5). In addition, NRF2 expression can be switched off by miR-153, miR-27a, miR-142-5p and miR144. The NRF2 partner Keap1 can be regulated by miR-200a, with the consequence that NRF2 is activated. Further activation of NRF2 can be achieved via miR-34a targeting sirtuin1 (Sirt1), which normally deacetylates NRF2 to promote its nuclear export (for reviews, see Cheng et al. 2013; Narasimhan et al. 2012). Another miRNA, miR-27 a/b, reduces prohibitin 1 and NRF2 (Yang et al. 2013) and leads to the appearance of non-alcoholic steatohepatitis-like symptoms and liver fibrosis. In addition, miR-433 decreases the expression of the major glutathione-synthesizing enzyme gamma-GCS (Espinosa-Diez et al. 2014) and contributes to oxidative stress. Furthermore, fibrotic kidneys have been found to display a loss of miR-30e, which, if present, would counteract EMT ( L. Jiang et al. 2013). Thus, miRNAs are important molecules at the point at which ROS signalling and fibrosis converge.

Other factors concerning the dissimilarities occurring in fibrosis are epigenetic changes. These are heritable traits that involve no changes in DNA sequences but where changes in DNA methylation and post-translational modifications of histones and other chromatin-associated proteins regulate transcription (Helin and Dhanak 2013). In particular, DNA methylation can be affected by ROS; oxidatively modified bases such as 8-oxo-2′-deoxyguanosine (8-oxodG) may inhibit the methylation of adjacent cytosines (Turk et al. 1995). The methylation of cytosine at its 5-position (5-methylcytosine [5mC]), sometimes called the “fifth base,” is associated with transcriptional silencing. As a consequence of 8-oxodG-inhibited methylation, the resulting hypomethylation results in transcriptional activation, whereby cells might gain new characteristics that promote various processes, among them fibrosis. On the other hand, ROS have also been linked to hypermethylation (Franco et al. 2008) and the down-regulation of repair genes such as O-6-methylguanine-DNA methyltransferase and MLH1 (mutL homologue 1; Ziech et al. 2011). Further, the hypermethylation of the phosphatase and tension homologue (PTEN) favours ERK and AKT signalling, cell proliferation and migration and the promotion of liver fibrosis (Bian et al. 2012). In addition, hypoxia has been associated with changes in global DNA methylation and, in particular, HIF-1α has been found directly to activate DNA methyltransferase 1 (DNMT1) and DNMT3B expression in cardiac fibroblasts. In agreement with this, the inhibition or genetic ablation of DNMT counteracts the hypoxia-induced expression of pro-fibrotic genes in cardiac tissue. Again, hypoxia-induced hypermethylation has been found in connection with the Thy-1 cell surface antigen promoter and appears to foster the development of a pro-fibrotic phenotype in human pulmonary fibroblasts (Robinson et al. 2012). Silencing of methylcytosine dioxygenase 3 has been found to contribute to bone-morphogenic-protein 7-induced reversal of kidney fibrosis attributable to 5-hydroxymethylcytosine formation and thus hypomethylation in the RAS protein activator promoter RASAL1 (Tampe et al. 2014). In addition, the hypermethylation and repression of Klf4 via Dnmt1 (Xiao et al. 2015) and the hypermethylation of methyl CpG binding protein 2 (Yang et al. 2013), RASAL1 (Tao et al. 2011), peroxisome proliferator-activated receptor (Zhao et al. 2013) and patched1 (Yang et al. 2013) also appear to promote fibrosis.

In addition to the above-mentioned factors, epigenetic modifications in antioxidant enzyme genes may also account for variations in the fibrotic process. Recent data point to the epigenetic silencing of the SOD2 gene, which may be a result of increased O_2_^•−^ levels, constituting a feed-forward mechanism promoting further epigenetic aberrancies (Hitchler et al. 2006; Teoh-Fitzgerald et al. 2012; Cyr et al. 2013). Thereby, O_2_^•−^ serves as a starting point for the generation of other ROS, with the consequence that metabolites such as NAD(+), S-adenosyl methionine and 2-oxoglutarate are altered. Because these substances are critical for sirtuins, histone methyltransferases, histone demethylases and DNA demethylases, these alterations immediately affect the epigenetic landscape (Cyr et al. 2013). Together, epigenetic aberrancies attributable to ROS might account for a number of variants seen in fibrotic phenotypes and could eventually be involved in the progression of cells from fibrotic to cancer phenotypes.

## Are antioxidants a treatment option in fibrosis?

The connection of fibrosis with ROS and oxidative stress implies that supplementation with nutrients or diets with antioxidants will, in addition to disease-specific therapies and the inhibition of TGF-β signalling, be beneficial. In general, this is not a new idea and part of a worldwide “antioxidant hype” that has been applied to other oxidative-stress-associated diseases such as cancer, type II diabetes and cardiovascular diseases (for a review, see Samoylenko et al. 2013). However, several studies have demonstrated that dietary supplementation with antioxidants does not have a positive influence but rather a negative effect on overall mortality; in particular, vitamin A, β-carotene and vitamin E increase mortality (Bjelakovic et al. 2007). Vitamin E has also been linked to prostate cancer (Klein et al. 2011). With respect to fibrosis, a prospective double-blind randomized placebo-controlled trial has been carried out among a total of 49 patients with non-alcoholic steatohepatitis receiving vitamins C and E (1000 mg and 1000 IU daily for 6 months, respectively) together with a diet limited to 1600 kcalories/day. Evaluation of the histological data from 45 patients finishing the study demonstrated no statistically significant differences in inflammation/necrosis between the vitamin group and the placebo group or within the vitamin or the placebo groups. Additionally, no significant difference in fibrosis was noted between the vitamin and the placebo groups. However, significant improvement in fibrosis was recorded within the group that received vitamins E and C but not in the placebo group (Harrison et al. 2003). Multicentre trials among patients suffering from idiopathic lung fibrosis have shown that antioxidant treatment has no or only a modestly beneficial effect (Demedts et al. 2005; Day 2008; Idiopathic Pulmonary Fibrosis Clinical Research Network et al. 2014). Further, vitamin supplementation has been shown to accelerate renal function decline in patients thought to have diabetic nephropathy (House et al. 2010). This lack of (or reduced) benefit not only applies to fibrosis but is also observed in cancer, type II diabetes and cardiovascular diseases and is known as the “antioxidant paradox” (Halliwell 2000; Hollman et al. 2011; Sheikh-Ali et al. 2011).

The reasons that antioxidants do not fulfil their expectations might be multiple and attributable; for example, to the wrong type/dose combinations, variations in treatment duration, poorly understood mechanisms of action and a lack of tests for compliance (for reviews, see Gorlach et al. 2015; Schmidt et al. 2015). However, recently, resveratrol, a natural antioxidant present in grapes and various berries, has been suggested as being of use in lung fibrosis, respiratory diseases such as asthma and chronic obstructive pulmonary disease. However, even if in vitro and animal data support resveratrol use, careful clinical patient characterization is needed, since resveratrol has been shown to exert opposite effects on the hypoxia-dependent expression of the breast cancer evidence marker PAI-1 in tumour vs. primary cells (Ganjam et al. 2015). Moreover, large clinical trials with resveratrol remain somewhat limited and further studies need to be performed to assess its real benefit (Conte et al. 2015). In particular, this seems to be important as regards dosage and the combination of antioxidants in the diet, since high doses of a single antioxidant in the diet have been shown to be harmful in smokers, whereas diets enriched with two antioxidants were safe in a randomised controlled trial in male smokers (Karlsen et al. 2011). Thus, antioxidant therapies for fibrosis and other diseases may still have a future but increased efforts with respect to research into ROS, antioxidants and disease pathogenesis in conjunction with large multicentre trials are required.

## Concluding remarks

All in all, ROS have an important role in fibrosis, in particular, in conjunction with the TGF-β1 signalling system. However, current knowledge represents just a basic understanding of the way that ROS contribute to fibrosis and details of the interplay with signalling pathways other than that involving TGF-β1 are largely lacking. Although the mechanisms involved show a causative role of ROS in fibrosis, they do not allow any precise conclusions or recommendations in terms of antioxidant therapy, which itself is somewhat questionable. At the same time, this lack of knowledge increases the demand for further understanding to be gained about the mechanisms linking the dynamics of ROS and antioxidant action and about the pathogenesis of fibrosis and its future therapies.
